# HypoxamiRs: the hidden architects of tissue adaptation in hypoxia

**DOI:** 10.1038/s41419-025-08091-0

**Published:** 2025-10-16

**Authors:** Virginia Egea

**Affiliations:** 1 Institute for Cardiovascular Prevention (IPEK), Ludwig-Maximilians-University (LMU), Munich, Germany; 2 German Center for Cardiovascular Research (DZHK), Partner Site Munich Heart Alliance, Munich, Germany

**Keywords:** Mechanisms of disease, Prognostic markers, Stress signalling

## Abstract

Hypoxia, or reduced oxygen availability, triggers a spectrum of adaptive responses across tissues, including angiogenesis, metabolic reprogramming, and modulation of survival pathways. Central to these adaptations are hypoxia-regulated microRNAs (miRNAs), hypoxamiRs, which fine-tune gene expression in a context-dependent manner. HypoxamiRs are transcriptionally regulated by hypoxia-inducible factors (HIFs), tissue-specific transcriptional programs, and microenvironmental cues, enabling precise responses to hypoxia. HypoxamiRs exhibit distinct expression profiles across tissues, reflecting their specialized roles. In ischemic tissue, they activate angiogenic and cytoprotective programs, while in metabolically active or malignant tissues, they rewire energy production and promote survival. This tissue specificity underlies their dual function as both regulators of physiological adaptation and drivers of pathology in chronic hypoxia. Increasingly, hypoxamiRs are being recognized as non-invasive biomarkers and therapeutic targets in diseases such as cancer, cardiovascular disorders, and fibrosis. Compared to canonical hypoxia pathways, hypoxamiRs offer a versatile and finely tunable layer of regulation. This review presents a unified framework in which hypoxamiRs emerge not merely as downstream effectors of HIF signaling but as integrative architects at the intersection of oxygen sensing, epigenetic remodeling, and cellular identity. Their coordinated regulatory functions make them promising tools for precision medicine in hypoxia-related diseases. Understanding how hypoxamiRs operate across tissues and pathologies may unlock new diagnostic and therapeutic strategies for complex, oxygen-sensitive conditions.

## Facts

- Hypoxia dynamically regulates the expression of specific miRNAs, termed hypoxamiRs, largely via transcription factors such as HIFs.
- HypoxamiRs exhibit tissue-specific expression patterns and influence key adaptive processes, including angiogenesis, metabolic reprogramming, apoptosis, and autophagy.
- miR-210 is a central hypoxamiR, consistently upregulated across cell types and conserved across species.
- Disruption of canonical miRNA biogenesis under hypoxia (e.g., via Dicer/Drosha inhibition or sequestration) reshapes the miRNA landscape in a tissue-dependent manner.
- HypoxamiRs are increasingly explored as non-invasive biomarkers and as therapeutic targets in diseases such as cancer, ischemia, and fibrosis.

## Open Questions

- Can non-canonical miRNA processing pathways compensate for suppressed Dicer/Drosha activity in hypoxic environments?
- How do hypoxamiRs interact with metabolic and epigenetic regulators to modulate tissue-specific hypoxia responses?
- What delivery platforms will be required to achieve safe, tissue-specific hypoxamiR modulation in clinical settings?

## The HIF-miRNA axis in hypoxic adaptation

**Fig. 1:**
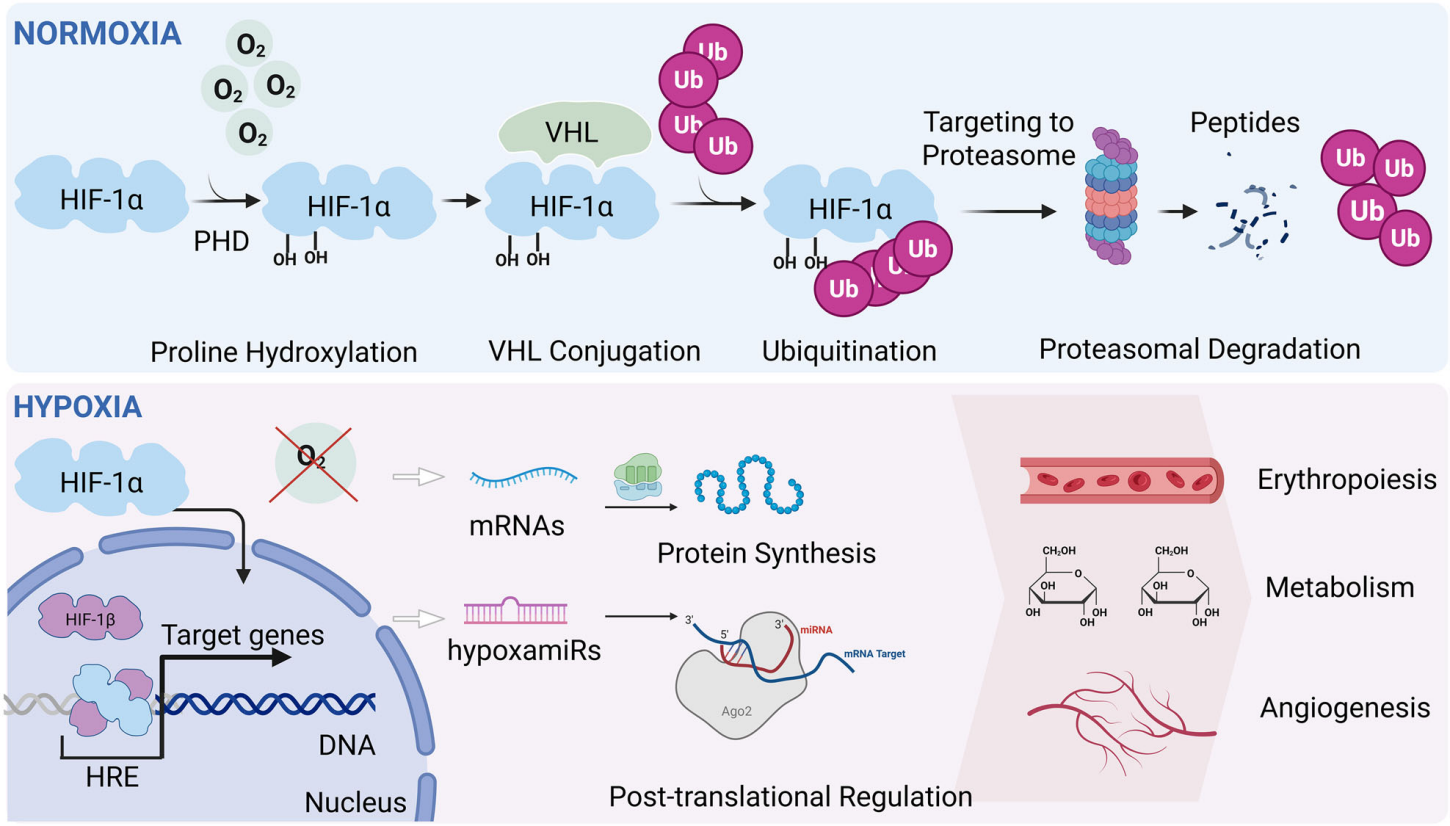
The HIF-1α signaling cascade. Under normoxic conditions, HIF-1α is hydroxylated at two proline residues. It then associates with VHL and is tagged with ubiquitin resulting in proteasomal degradation. Under hypoxic conditions, HIF-1 alpha translocates to the cell nucleus and associates with HIF-1β. This complex then binds to the HRE region of the DNA resulting in the transcription of RNAs including a group of miRNAs (so-called hypoxamiRs) that are involved in a multitude of processes including erythropoiesis, glycolysis, and angiogenesis.

Oxygen (O_2_) is a key substrate for cellular metabolism and energy production [1]. In a variety of physiological and pathological states, organisms encounter insufficient O_2_ availability, or hypoxia. In order to cope with this stress, evolutionarily conserved responses are engaged. In mammals, the primary transcriptional response to hypoxic stress is mediated by the Hypoxia-inducible factors (HIFs) [2, 3]. The HIFs are members of the basic helix-loop-helix/Per-Arnt-Sim (bHLH/PAS) family of transcription factors (TFs) that function as heterodimers composed of an oxygen-labile α subunit and a constitutively-expressed β subunit. Mammalian species possess three α isoforms: HIF-1α, HIF-2α, and HIF-3α. HIF-1α and, to a lesser extent, HIF-2α, are the best characterized and most structurally similar, while HIF-3α‘s function is less clear as it exists as multiple splice variants, some of which exert inhibitory transcriptional control over the other isoforms [4]. In normoxic conditions, HIF proteins have a short half-life of <5 min, being targeted for ubiquitination and proteasomal degradation by the von Hippel-Lindau tumor suppressor protein after hydroxylation of specific proline residues within an oxygen-dependent domain by HIFα-specific prolyl hydroxylases (PHDs) (Fig. 1). Under hypoxic conditions, oxygen is limited or unavailable to PHDs as a co-substrate, and HIF-α subunits are stabilized and subsequently translocate to the nucleus where they form heterodimers with the constitutively expressed β subunits. These heterodimers bind to hypoxia-response elements (HREs) with a core RCGTG motif and to ancillary sequences that recruit additional TFs (Fig. 1) [2]. More than 70 genes have been identified as bona fide direct HIF-1α targets containing an HRE, and more than 200 genes have been identified using microarray as being affected by hypoxia and therefore direct or indirect targets of HIFs [5]. Additionally, HIF-1α is a potent regulator of microRNA (miRNA) expression, and its nuclear translocation induces the transcription of over 90 different miRNAs, also called hypoxamiRs, that in turn are potent regulators of many aspects of the hypoxic behavior [6]. However, not all hypoxamiRs are regulated exclusively by HREs. Several other TFs are activated during hypoxia and may regulate hypoxamiR expression independently or synergistically with HIFs [7]. Notable examples include nuclear factor-κB (NF-κB), which integrates inflammatory and hypoxic signals and can directly induce miR-155 and miR-210 [8]; c-Myc, which modulates the expression of miR-17 ~ 92 and interacts with HIF to coordinate metabolic reprogramming [9]; and p53, which responds to severe hypoxia by regulating pro-apoptotic miRNAs such as miR-34a [10]. Additionally, TFs such as cAMP response element-binding protein (CREB) and E26 transformation-specific-1 (Ets-1) have been shown to bind hypoxamiR promoters under stress conditions, further expanding the regulatory landscape beyond HIF-dependency [11].

miRNAs as a class of small (~22 nucleotides long), non-coding RNAs, can modulate gene expression through interference with mRNA translation [12]. They are key factors in the regulation of gene expression in both normal and neoplastic tissue, and they are being actively explored as prognostic indicators, biomarkers, and diagnostic and therapeutic targets [13]. Many miRNAs are highly conserved across species, especially within the so-called “seed” region in their sequence that serves as the main determinant of target specificity [13].

miR-210 is surely the most extensively studied hypoxamiR [14]. In a manner similar to conventional genes, HIF-1α directly binds to an HRE located on the proximal promoter of miR-210 [15]. The conservation of this HRE site across different species, when comparing the core promoter of miR-210, underscores the importance of hypoxia and HIFs in regulating the expression of miR-210 throughout evolution [16]. Beyond transcriptional control, miRNAs are subject to complex layers of post-transcriptional regulation during hypoxia, which also contribute to their tissue-specific expression [17].

**Fig. 2:**
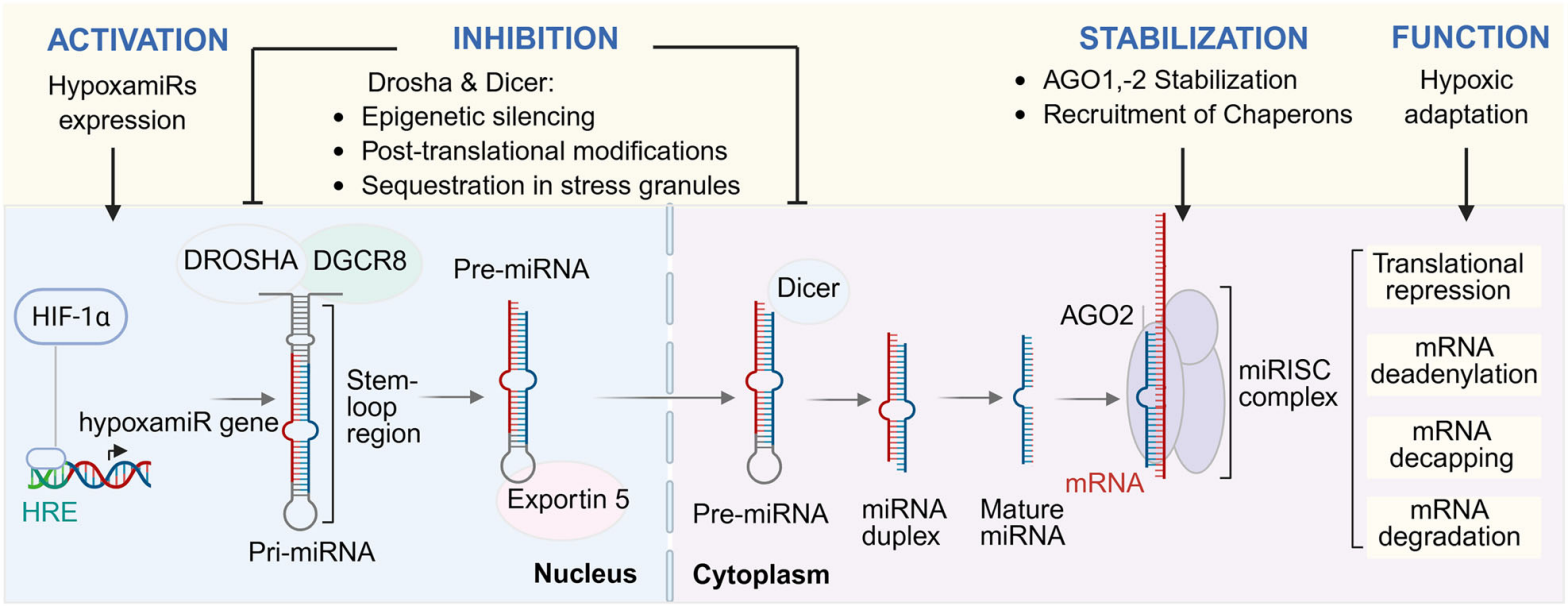
Biogenesis and regulation of hypoxamiRs under hypoxic conditions. Hypoxia induces the transcription of hypoxamiR genes via HIF-1α binding to hypoxia-responsive elements (HREs). Primary miRNAs (pri-miRNAs) are processed in the nucleus by the microprocessor complex, including Drosha and DGCR8, into precursor miRNAs (pre-miRNAs), which are exported to the cytoplasm by Exportin-5. Further processing by Dicer generates the miRNA duplex, from which the mature miRNA is loaded into the Argonaute 2 (AGO2) protein to form the miRISC complex. Hypoxia modulates this pathway at multiple levels: **(1) Activation**—increased transcription of hypoxamiRs; **(2) Inhibition**—through epigenetic silencing, post-translational modifications, or sequestration of processing components as Drosha and Dicer in stress granules; **(3) Stabilization**—via enhanced AGO2 stabilization and chaperone recruitment; and **(4) Function**—the miRISC complex mediates translational repression and mRNA decay mechanisms to enable cellular adaptation to low oxygen availability.

As depicted in Fig. 2, hypoxia can downregulate key components of the miRNA biogenesis machinery, such as Drosha and Dicer, through transcriptional repression [18]. HIF-1α has been shown to repress Dicer expression in certain cancer cells by recruiting transcriptional repressors such as histone deacetylases (HDACs) or interacting with co-repressors like SIN3A, which modify chromatin structure and reduce accessibility at the DICER1 promoter [19]. Additionally, epigenetic modifications like promoter methylation can silence DROSHA expression, such as CpG island hypermethylation observed in hypoxia-exposed glioblastoma cells, which leads to reduced Drosha transcription and impaired pri-miRNA processing at the nuclear level [20]. Drosha downregulation limits the conversion of pri-miRNAs to pre-miRNAs, while reduced Dicer levels impair the maturation of pre-miRNAs into functional miRNAs, thereby shifting the miRNA landscape toward hypoxia-adaptive profiles. In addition, post-translational modifications (including phosphorylation, acetylation, or ubiquitination) can alter the enzymatic activity, localization, or stability of these proteins, further modulating miRNA processing under low-oxygen conditions [18]. Hypoxia has also been shown to induce the sequestration of Drosha and Dicer into subcellular structures such as stress granules, reducing their access to RNA substrates and further dampening global miRNA processing [21]. As a result, the biogenesis of specific miRNAs can be either selectively enhanced or suppressed, contributing to tissue-specific and condition-specific responses (Fig. 2) [22, 23]. Additionally, RNA-binding proteins such as HuR and Lin28, which interact with precursor or mature miRNAs, can further modulate miRNA stability and activity in a tissue-specific manner [24]. HuR can bind to pri- or pre-miRNAs and either stabilize them or inhibit their processing, depending on the cellular context, while LIN28A promotes HIF1α protein stabilization independently of the downregulation of miRNA let-7, which is also directly mediated by LIN28A [25, 26].

Several mechanistic questions remain open. Given the recurrent suppression of Dicer under hypoxic conditions, it is plausible that certain hypoxamiRs may rely on non-canonical processing routes to maintain regulatory function [27]. Similarly, the sequestration of Drosha and Dicer into stress granules raises the possibility of subcellular compartmentalization as a dynamic switch in miRNA biogenesis. Another emerging hypothesis is that hypoxamiRs engage in context-specific feedback with metabolic sensors or epigenetic regulators, forming circuits that fine-tune tissue responses beyond HIF signaling. Intriguingly, recent evidence suggests that specific hypoxamiRs may translocate into the nucleus during hypoxic stress, where they can associate with chromatin-modifying complexes or long non-coding RNAs to modulate transcriptional programs in a miRNA-dependent but Dicer-independent manner [28]. This spatial re-routing adds a new layer of regulatory potential, positioning hypoxamiRs not only as post-transcriptional repressors, but also as modulators of nuclear gene expression. Exploring these mechanisms could uncover novel dimensions of RNA-based regulation in hypoxia adaptation.

## Concept of tissue-specific regulation

Tissue-specific regulation of miRNAs, particularly hypoxamiRs, plays a pivotal role in fine-tuning cellular responses to hypoxia across diverse tissue types. Rather than acting as uniformly expressed effectors, hypoxamiRs exhibit distinct expression patterns that align with the physiological demands and molecular architecture of each tissue. Their regulatory effects often intersect with HIF signaling, either modulating its activity or reinforcing tissue-specific gene expression programs that enable precise adaptation to hypoxic stress. HypoxamiRs are expressed under both physiological and pathological conditions, where they contribute to either homeostatic adaptation or the progression of hypoxia-related diseases such as ischemia and cancer. As a result, identical hypoxic stimuli can yield divergent cellular outcomes, shaped by the presence and function of context-dependent hypoxamiRs such as miR-210, miR-21, and miR-126. This variability reflects not only differences in miRNA abundance, but also in the composition of target mRNAs, transcriptional regulators, and epigenetic landscapes across tissues. Ultimately, the tissue-specific behavior of hypoxamiRs emerges from a network of interdependent factors that includes cell lineage, metabolic state, inflammatory cues, and local microenvironmental signals.

## Tissue-specific transcriptional regulation

The regulation of hypoxamiRs is tightly controlled by tissue-specific TFs that align miRNA expression with the physiological and pathological demands of each tissue. This regulation ensures that hypoxia triggers adaptive mechanisms suited to the functional requirements of different organs. Beyond HIFs, several other TFs are activated during hypoxia and may regulate hypoxamiR expression independently or synergistically with HIFs [7]. In cardiac tissue, TFs such as HIF-1α and nuclear NF-κB coordinate the expression of miRNAs like miR-21 and miR-210, promoting angiogenesis, reducing apoptosis, and protecting cardiomyocytes during ischemia [29, 30]. In the brain, hypoxia activates TFs such as CREB, which regulate hypoxamiRs like miR-155 and miR-124 to mitigate inflammation, enhance neuronal survival, and support neurogenesis [31]. Vascular endothelial cells rely on hypoxamiRs such as miR-126 and miR-21, regulated by TFs like Ets-1, to promote angiogenesis and vascular remodeling essential for tissue oxygenation during ischemia [11].

The regulation of hypoxamiRs is shaped by intricate interactions between HIFs and tissue-specific TFs also in cancer. For example, in tumor cells, the interplay between p53 and HIF-1α modulates opposing miRNA networks, with p53 inducing tumor-suppressive miR-34a and HIF-1α promoting pro-survival miR-210 [32]. These coordinated responses allow tissues to balance survival and apoptosis based on the severity of hypoxic stress.

## Physiological and pathological hypoxamiRs

Under normal circumstances, hypoxamiRs help cells survive by regulating processes like apoptosis, angiogenesis, and metabolic shifts. However, in certain pathological conditions, such as chronic hypoxia seen in cancer, neurodegenerative diseases, and ischemic injuries, hypoxamiRs can become pathological. In these contexts, their dysregulation contributes to disease progression (Table 1) [33].

**Table 1:** Functional and pathological roles of validated miRNAs across tissues.

miRNA	Tissue/Context	Target Genes	Function (Physiological)	Function (Pathological)	Citation
**miR-210**	Hypoxic tissues (brain, heart, tumors)	ISCU, SDHD, HIF-1α	Promotes angiogenesis	Tumor proliferation and resistance in cancers.	[16, 117]
**miR-155**	Immune cells (macrophages, T cells)	SOCS1, SHIP1, NF-κB	Modulates immune response	Drives inflammation in rheumatoid arthritis, atherosclerosis, and neurodegeneration	[31]
**miR-21**	Brain, heart, tumors	PTEN, PDCD4, TGF-β pathways	Regulates apoptosis and cell survival	Promotes fibrosis and oncogenesis in cancers; exacerbates neuroinflammation in neurodegenerative diseases	[58, 124]
**miR-124**	CNS (neurons, astrocytes)	STAT3, BACE1, PTPN1	Supports neuronal differentiation	Implicated in Alzheimer’s disease and recovery mechanisms post-stroke	[125]
**miR-126**	Endothelial cells	VEGFA, SPRED1, PIK3R2	Maintains vascular integrity	Dysregulated in diabetes; contributes to vascular complications	[39, 44, 46]
**miR-27a**	Liver, cancer tissues	CYP1B1, ABCG2	Regulates metabolic enzymes	Contributes to drug resistance in cancers	[126]
**miR-29**	Pancreas, fibrotic tissues	Collagen, MMP2, Wnt pathway	Regulates ECM remodeling	Dysregulation associated with fibrosis and diabetic complications	[127]

This table integrates the expression patterns, physiological roles, and pathological contributions of validated hypoxamiRs across various tissues and disease contexts.

**Fig. 3:**
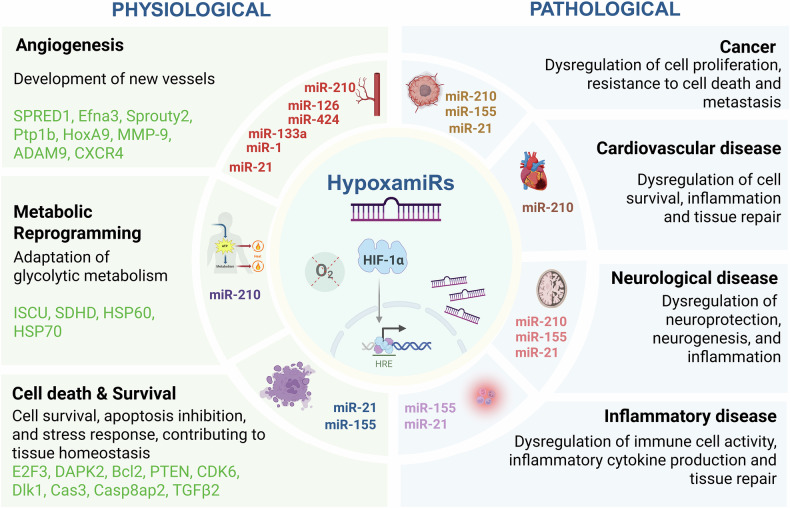
HypoxamiRs in physiological and pathological processes. HypoxamiRs help cells adapt to low-oxygen conditions by regulating a wide range of processes, from angiogenesis and metabolic reprogramming to cell survival and immune responses. Their importance extends beyond basic physiological processes to play a significant role in the pathogenesis of diseases like cancer, cardiovascular diseases, neurological disorders, and chronic inflammatory conditions.

While miR-210 initially helps cells adapt to acute hypoxia by promoting survival and tissue repair, sustained or excessive expression in chronic hypoxia can lead to maladaptive cellular responses. This prolonged activation of hypoxamiRs can interfere with normal cellular functions, promoting tumor progression or exacerbating neuronal damage. In pathological states, the imbalance in their target gene regulation can result in abnormal cell survival, contributing to tumor growth or neuronal dysfunction. For instance, the inhibition of pro-apoptotic genes or tumor suppressors by hypoxamiRs may allow damaged cells to evade death, further driving disease progression (Fig. 3) [33].

## Emerging principles of hypoxamiR function across tissues

**Fig. 4:**
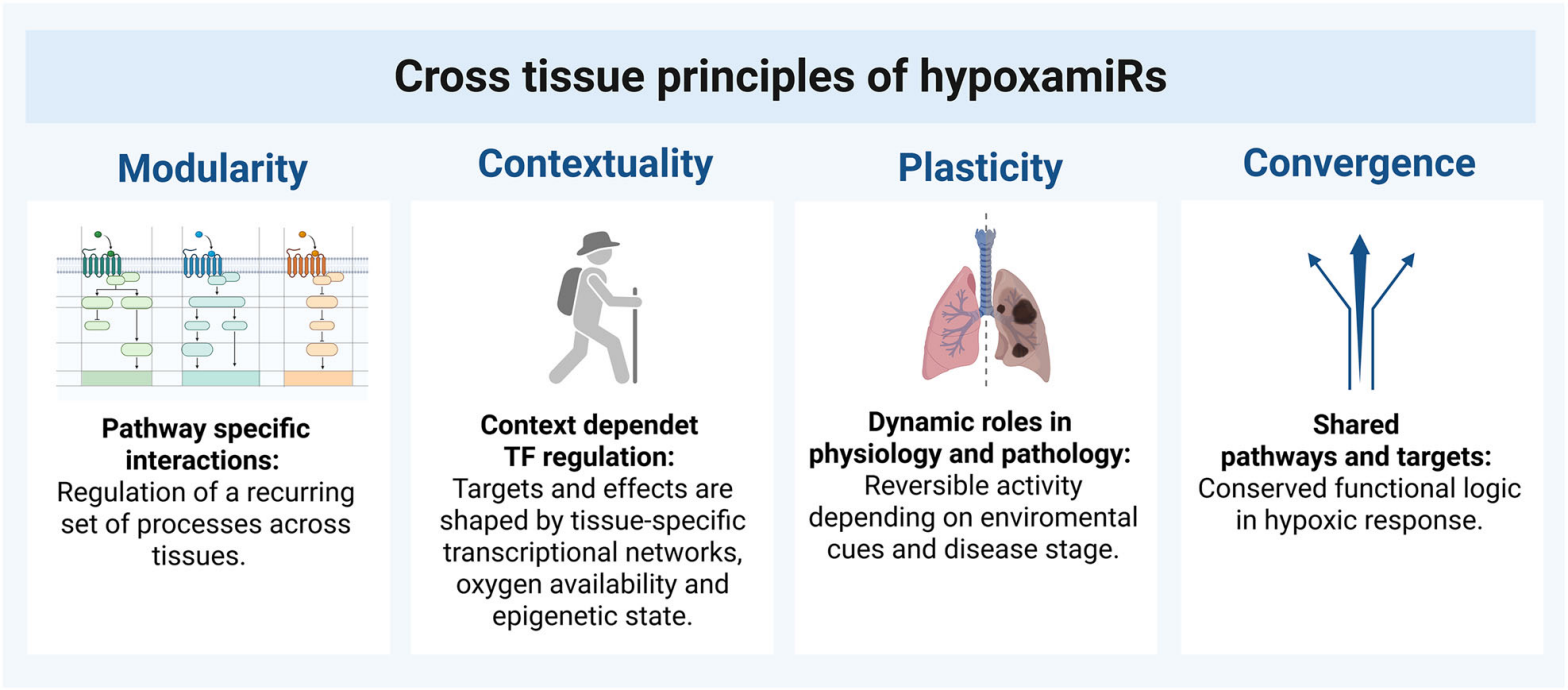
Conceptual framework summarizing cross-tissue principles of hypoxamiR function.

As research into hypoxia-regulated microRNAs advances, it becomes clear that their functions are governed by a set of emerging principles that transcend individual tissues or disease contexts. These principles: modularity, contextuality, plasticity, and convergence, offer a conceptual framework for understanding the dynamic and adaptive nature of hypoxamiR regulation in complex biological systems (Fig. 4).

Modularity refers to the ability of hypoxamiRs to engage in discrete, pathway-specific interactions within particular physiological or pathological modules. Rather than being universally expressed or acting through uniform pathways, hypoxamiRs often regulate specific gene networks tailored to a tissue’s structural and functional needs. For example, miR-210 modulates angiogenesis in ischemic tissues, while in the skeletal system it influences osteogenic differentiation, illustrating how the same molecule can be deployed in modular fashion across different physiological settings.

Building on this modularity, contextuality reflects the capacity of hypoxamiRs to exert different regulatory effects depending on the local cellular environment. The expression, stability, and targets of hypoxamiRs are profoundly influenced by the interplay of tissue-specific TFs, chromatin architecture, and signaling cross-talk. For instance, while HIF-1α remains a master upstream regulator, additional transcriptional inputs such as NF-κB, p53, or CREB enable tissue-specific expression patterns. This context-dependence allows hypoxamiRs to fine-tune responses based on the severity, duration, and microenvironmental features of hypoxia.

Plasticity captures the ability of hypoxamiRs to adapt their roles during physiological versus pathological states. In acute hypoxia, they often act as cytoprotective agents by promoting angiogenesis, suppressing apoptosis, or shifting metabolism toward glycolysis. However, under chronic hypoxic conditions, the same hypoxamiRs may contribute to disease progression by fostering fibrosis, immune dysregulation, or oncogenic transformation. This duality underscores the need to consider both timing and context when evaluating hypoxamiR function, especially in therapeutic design.

Finally, convergence describes how different hypoxamiRs tend to target overlapping signaling hubs involved in adaptation to hypoxia. Central pathways such as PI3K/Akt, VEGF, Notch, and mitochondrial metabolism are modulated by multiple hypoxamiRs, often acting in parallel or redundantly. Moreover, hypoxia also affects the core miRNA processing machinery (e.g., Drosha, Dicer), RNA-binding proteins (HuR, LIN28), and stress-related modulators (e.g., SIN3A-HDAC1), adding additional layers of regulation that converge on miRNA biogenesis and activity.

Together, these emerging principles (modularity, contextuality, plasticity, and convergence) offer a cohesive framework for interpreting hypoxamiR behavior across tissues (Fig. 4).

## Tissue-specific hypoxamiRs and their roles

The tissues selected for detailed analysis in this review (cardiovascular, neurological, pulmonary, skeletal, adipose, and cancer) were chosen based on (i) their high metabolic demand, (ii) the frequency and physiological relevance of hypoxic episodes, and (iii) the availability of mechanistic studies linking hypoxamiRs to disease progression or tissue adaptation. While hypoxia is also critically involved in other systems such as the renal and gastrointestinal tracts, we prioritized systems with a strong translational body of literature to maintain scope and focus. Future reviews may expand on hypoxamiR functions in these additional organs.

## Cardiovascular system

The cardiovascular system exemplifies a dynamic battlefield where hypoxamiRs orchestrate adaptive survival tactics. Through fine control of angiogenesis, metabolic shifts, and cellular stress responses, they help preserve tissue perfusion in the face of ischemic injury [34, 35].

### miR-210

Among the most consistently upregulated hypoxamiRs, miR-210 integrates multiple layers of endothelial adaptation to hypoxia. Under hypoxic conditions, miR-210 targets E2F transcription factor 3 (E2F3) [36], a transcription factor involved in cell cycle regulation. It also promotes angiogenesis through repression of Efna3 (ephrin-A3), thereby facilitating endothelial migration and sprouting [37]. In addition, miR-210 suppresses protein tyrosine phosphatase 1B (Ptp1b), amplifying vascular endothelial growth factor (VEGF) signaling and vascular responsiveness to hypoxic stimuli [38]. Further supporting vascular remodeling, miR-210 downregulates HoxA9, a transcription factor essential for endothelial differentiation [16]. At the metabolic level, miR-210 adjusts mitochondrial function under hypoxia by targeting iron-sulfur cluster scaffold protein (ISCU), which impacts mitochondrial respiration and promotes a glycolytic shift, ensuring energy production in oxygen-deprived environments. It also inhibits succinate dehydrogenase complex subunit D (SDHD), reducing reactive oxygen species (ROS) production and protecting endothelial cells from oxidative damage during hypoxia [15].

### miR-126

miR-126 serves as another critical regulator of endothelial integrity under hypoxia. It reinforces VEGF signaling by targeting Sprouty-related EVH1 domain-containing protein 1 (Spred1), and activating the phosphoinositide 3-kinase (PI3K)/Akt signaling pathway, both of which promote endothelial survival and angiogenesis [35]. By modulating vascular endothelial growth factor receptor 2 (VEGFR2) and downstream targets, miR-126 supports neovascularization in ischemic tissues [17, 39, 40]. Additionally, it regulates matrix metalloproteinase-9 (MMP-9), metalloproteinase domain-containing protein 9 (ADAM9), and C-X-C chemokine receptor type 4 (CXCR4), promoting endothelial migration and matrix remodeling [41–43]. Its influence extends to sphingosine-1-phosphate receptor 1 (S1PR1), and delta-like 1 homolog (DLK1), two factors implicated in endothelial differentiation and vascular permeability [44]. Notably, miR-126 also exhibits a non-canonical role by binding to and inhibiting caspase-3, directly suppressing apoptosis [17, 45]. Finally, miR-126 downregulates forkhead box O1 (FOXO1), further supporting endothelial cell survival and maintaining vascular integrity in hypoxic environments [46].

### MiR-424

Closely mirroring the vascular effects of miR-126, miR-424 also enhances VEGF signaling via Spred1 suppression and supports endothelial survival through PI3K/Akt activation [47]. Beyond survival, it promotes endothelial proliferation by repressing cell division protein kinase 6 (CDK6) and E2F transcription factor 3 (E2F3), and fine-tunes angiogenesis via Notch signaling modulation [48]. miR-424 further enhances endothelial cell survival under hypoxia by regulating sirtuin-1 (SIRT1), a key player in oxidative stress response [49]. In addition to promoting angiogenesis, miR-424 downregulates transforming growth factor beta-2 (TGF-β2), reducing fibrosis and preventing excessive vascular remodeling, which helps maintain healthy blood vessels in ischemic tissues [50]. Finally, by targeting p27^kip1, a cyclin-dependent kinase inhibitor, miR-424 promotes endothelial cell proliferation and migration, which are vital for effective angiogenesis and vascular repair during hypoxic stress [50].

### miR-1 and miR-133a

These muscle-specific miRNAs exemplify the coordinated hypoxic response in cardiomyocytes. Though co-transcribed, they diverge in function: miR-1 modulates cardiac excitability and apoptosis, whereas miR-133a controls fibrosis and proliferation [51]. Under hypoxia, miR-1 downregulation permits upregulation heat shock protein 60 (HSP60) and heat shock protein 70 (HSP70) [52], enhancing mitochondrial resilience, and modulates ion channel activity via connexin 43 (GJA1) and potassium channel inwardly rectifying subfamily J member 2 (KCNJ2) to stabilize conduction [53]. In contrast, miR-133a suppresses fibrotic mediators as connective tissue growth factor (CTGF), alpha-1 type I collagen (COL1A1) and cytoskeletal regulators (RhoA), limiting maladaptive remodeling [54]. Their opposing roles in apoptosis, underscore a dynamic balance tailored to ischemic demands, while miR-1 promotes apoptosis by targeting Bcl-2, miR-133a counteracts this by suppressing caspase-9 and other apoptotic mediators as death associated protein kinase 2 (DAPK2), creating a balance that supports cell survival and prevents excessive cell loss in ischemic tissues [55, 56].

Together, miR-1 and miR-133a regulate cardiac excitability, myocyte survival, fibrosis, and remodeling during hypoxic stress, highlighting their complementary and critical roles in maintaining cardiovascular function and resilience under oxygen-deprived conditions.

### miR-21

Acting at the interface of survival, inflammation, and fibrosis, miR-21 modulates multiple key targets during hypoxia. One of miR-21´s key functions is the inhibition of phosphatase and tensin homolog (PTEN), a negative regulator of the PI3K/Akt pathway [57]. This inhibition enhances Akt signaling, promoting endothelial cell survival and proliferation under hypoxic stress. miR-21 also suppresses Sprouty2, relieving its inhibition of the pathway, which further supports angiogenesis and vascular remodeling. miR-21 contributes to extracellular matrix remodeling by targeting TIMP3 (tissue inhibitor of metalloproteinase-3) [58]. This regulation facilitates matrix degradation and endothelial cell migration, crucial for angiogenesis during hypoxia. Additionally, miR-21 downregulates programmed cell death 4 (PDCD4), reducing apoptosis and supporting endothelial cell viability in ischemic environments [59]. Under hypoxia, miR-21 plays a role in anti-inflammatory signaling by modulating the NF-κB pathway, which helps mitigate inflammatory damage in vascular tissues. Through regulation of TGF-β components such as Smad7, miR-21 helps strike a balance between beneficial vascular remodeling and fibrosis, emphasizing its dual role in vascular repair and pathology [59].

## Neurological system

In the brain, where oxygen availability tightly governs function and survival, hypoxamiRs act as rapid-response regulators. They fine-tune neuroprotection, inflammation, and repair mechanisms under both acute and chronic hypoxic stress.

### miR-210

Among the most prominent hypoxamiRs in the central neural system (CNS), miR-210 supports neuronal survival through both metabolic and anti-apoptotic mechanisms. miR-210 targets ISCU, which diminishes oxidative phosphorylation and limits ROS production, protecting neurons from oxidative damage. Additionally, miR-210 promotes neuronal survival by suppressing pro-apoptotic factors like caspase-8-associated protein 2 (Casp8ap2) and stabilizing HIF-1α indirectly through the inhibition of glycerol-3-phosphate dehydrogenase 1-like (GPD1L). This stabilization amplifies the hypoxic response, further enhancing cell survival mechanisms [60]. Beyond its role in survival, miR-210 facilitates angiogenesis by repressing EFNA3 [37], thereby supporting vascular remodeling and improving oxygen supply to hypoxic neural tissues. It also promotes neurogenesis by enhancing the survival and differentiation of neural stem/progenitor cells in low oxygen environments, which is particularly critical in repair mechanisms following ischemic brain injury. Moreover, miR-210 modulates inflammation by influencing glial cell activity, balancing the neuroinflammatory response to minimize secondary damage. Its multifaceted role positions it as a key player in ischemic conditions like stroke, where it aids in protecting neurons and stimulating recovery. However, in some neurodegenerative diseases, excessive miR-210 expression may contribute to mitochondrial dysfunction and exacerbate oxidative stress, underscoring its dual role in both adaptation and pathology [61, 62].

### miR-155

Under hypoxic stress, miR-155 plays a dual role in the CNS, contributing to both damage and repair processes depending on the context. miR-155 directly targets suppressor of cytokine signaling 1 (SOCS1), a negative regulator of the inflammatory JAK/STAT pathway [63, 64]. This suppression leads to enhanced cytokine production and microglial activation, amplifying neuroinflammation. While inflammation is necessary for initiating repair processes, excessive inflammation driven by miR-155 can exacerbate neuronal injury and increase tissue damage [65]. In addition to its role in inflammation, miR-155 modulates apoptotic pathways by targeting FOXO3a and B-cell lymphoma 2 (Bcl-2), tipping the balance toward neuronal apoptosis under severe hypoxia [66]. However, miR-155 also targets peroxisome proliferator-activated receptor gamma (PPARγ), which is involved in lipid metabolism and inflammation resolution, suggesting that it has a context-dependent function in regulating inflammation and metabolic adaptation in ischemic brain tissue [67].

## Pulmonary system

As the frontline of oxygen exchange, the pulmonary system is another key player in hypoxic responses, with hypoxia contributing to diseases like chronic obstructive pulmonary disease (COPD) [68], pulmonary hypertension, and acute respiratory distress syndrome (ARDS) [69]. HypoxamiRs regulate vascular remodeling, inflammation, and cell survival, which are central to the adaptation of the lungs to low-oxygen conditions.

### miR-210

Also in pulmonary cells, miR-210 acts as a central regulator of pulmonary adaptation to hypoxia, balancing metabolism, survival, and inflammation. miR-210 modulates mitochondrial metabolism by targeting ISCU1/2 reducing ROS generation and minimizing oxidative damage under hypoxic conditions. miR-210 also promotes angiogenesis in the lungs by targeting Ephrin-A3 [14]. This function is critical in hypoxia-induced pulmonary vascular remodeling, a hallmark of diseases such as pulmonary arterial hypertension (PAH). Another significant target of miR-210 is autophagy-related gene 7 (ATG7) [70]. Inhibition of autophagy by miR-210 prevents excessive cell degradation and helps maintain pulmonary cell viability under oxygen deprivation. In parallel, miR-210 influences inflammation by targeting NF-κB pathway modulators, reducing excessive inflammatory responses in pulmonary tissues during hypoxic stress. miR-210 also contributes to smooth muscle cell proliferation and vascular remodeling by regulating programmed cell death protein 4 (PDCD4) and other apoptotic mediators. Taken together, these actions enhance vascular resistance and contribute to the pathophysiology of hypoxia-driven pulmonary diseases [71].

### miR-155

miR-155 is a key immune regulator in the hypoxic lung, with both protective and damaging effects. In hypoxic pulmonary tissues, miR-155 modulates inflammation by targeting SOCS1 [63, 64]. Suppression of SOCS1 amplifies inflammatory cytokine production, enhancing immune responses. While this can be protective initially, excessive inflammation driven by miR-155 may exacerbate tissue damage, contributing to conditions like acute lung injury (ALI) or chronic obstructive pulmonary disease (COPD). miR-155 also regulates pulmonary vascular remodeling, by targeting bone morphogenetic protein receptor type 2 (BMPR2), miR-155 disrupts signaling pathways that maintain vascular homeostasis, promoting smooth muscle cell proliferation and vascular resistance [72]. Additionally, miR-155 influences endothelial cell behavior by suppressing eNOS (endothelial nitric oxide synthase), impairing vasodilation and contributing to hypoxia-induced vascular dysfunction [73]. In the context of oxidative stress, miR-155 targets FOXO3a, a transcription factor involved in antioxidant defense. This regulation impacts the balance between ROS production and clearance, further contributing to hypoxia-induced pulmonary damage [66, 74].

Finally, its regulation of Src homology 2 domain-containing phosphatase 2 (SHP2), an important modulator of macrophage polarization, links miR-155 to both the initiation and resolution of pulmonary inflammation [75, 76].

### miR-126

Primarily expressed in endothelial cells, miR-126 preserves vascular integrity and promotes adaptive angiogenesis in the hypoxic lung. miR-126 targets SPRED1, a negative regulator of the RAS/RAF/ERK pathway, enhancing pro-angiogenic signaling, endothelial proliferation, and capillary growth. Additionally, by suppressing phosphoinositide-3-kinase regulatory subunit 2 (PIK3R2), it activates the PI3K/Akt pathway, supporting endothelial survival and nitric oxide production, critical for maintaining vascular function during hypoxia [77]. Beyond angiogenesis, miR-126 reduces hypoxia-induced inflammation by targeting vascular cell adhesion molecule-1 (VCAM-1) and intercellular adhesion molecule-1 (ICAM-1), limiting leukocyte adhesion and endothelial inflammation. It also indirectly regulates smooth muscle cell behavior, curbing excessive proliferation and vascular remodeling, which are hallmarks of pulmonary arterial hypertension (PAH) [78, 79]. In summary, miR-126 ensures vascular homeostasis and mitigates inflammation during hypoxia, making it a key therapeutic target for hypoxia-related pulmonary conditions such as PAH, acute respiratory distress syndrome (ARDS), and chronic obstructive pulmonary disease (COPD).

## Skeletal system

Bone is not a passive structure but a metabolically active tissue that responds dynamically to oxygen availability. Hypoxia influences key processes such as bone formation, remodeling, and repair, and hypoxamiRs act as important molecular regulators of these responses.

### miR-210

miR-210 plays a central role in hypoxic adaptation of the skeletal system by integrating osteogenic, angiogenic, and metabolic pathways. Induced by HIF-1α, miR-210 enhances osteoblast differentiation by targeting dual specificity phosphatase 1 (DUSP1), thereby promoting MAPK signaling and bone matrix production [80]. In parallel, it supports angiogenesis through repression of EFNA3, facilitating endothelial cell migration and vascularization within bone tissue [80]. Additionally, miR-210 regulates osteoclastogenesis through HIF-1α and Notch signaling, balancing bone resorption and formation [80, 81]. In summary, miR-210 plays a vital role in bone adaptation to hypoxia by promoting osteoblast function, angiogenesis, and remodeling, crucial for maintaining skeletal health in hypoxic conditions.

### miR-148a

miR-148 is involved in the regulation of skeletal system functions, particularly during hypoxic conditions. Although less studied in the context of hypoxia, emerging evidence suggests its role in bone remodeling, osteogenesis, and cellular response to low oxygen levels. Under hypoxia, miR-148 targets DNA methyltransferase 1 (DNMT1) [82], leading to alterations in gene expression that favor osteoblast differentiation and function. This regulation supports bone formation and remodeling, essential during periods of low oxygen availability. miR-148 also influences bone resorption by modulating [83] NF-κB signaling, which is critical for osteoclast differentiation. Additionally, miR-148 may contribute to angiogenesis in bone tissue, supporting vascular supply necessary for bone health under hypoxic stress. While its exact mechanisms in the skeletal system require further exploration, miR-148 appears to play a key role in maintaining bone homeostasis during oxygen deprivation [83]. In summary, miR-148 supports osteogenesis, bone remodeling, and angiogenesis in the skeletal system under hypoxia, making it a potential regulator in bone health during conditions of low oxygen availability.

## Adipose tissue

Adipose tissue is a metabolically dynamic organ that undergoes profound changes in response to hypoxia, especially in the context of obesity, insulin resistance, and thermoregulation. HypoxamiRs play tissue-specific roles in brown adipose tissue (BAT) and white adipose tissue (WAT), shaping divergent adaptive strategies [84].

### miR-210

miR-210 acts as a central hypoxia-responsive regulator in both brown and white fat, though with contrasting functional outcomes.

In BAT, hypoxia impairs differentiation and thermogenic capacity, with miR-210 playing a central regulatory role [85]. miR-210 disrupts brown adipocyte differentiation and lipid storage. This blunting of BAT activity under hypoxic stress may contribute to metabolic dysfunction, particularly in obesity where oxygen availability is chronically reduced. In contrast, miR-210 plays a different role in WAT, where it supports lipid accumulation and adaptation to hypoxic stress [86]. In white adipocytes, miR-210 enhances lipogenesis and promotes metabolic flexibility, prioritizing fat storage and survival pathways over energy expenditure [87]. These tissue-specific effects highlight the dual role of miR-210 as both a suppressor of energy dissipation in BAT and a facilitator of energy storage in WAT. Understanding this dichotomy is critical for developing targeted interventions in hypoxia-associated metabolic disease.

### miR-21

In WAT, hypoxia-induced miR-21 contributes to adipose tissue remodeling by repressing pro-apoptotic and anti-angiogenic genes such as PTEN and PDCD4. This facilitates cell survival and promotes a microenvironment that supports expansion and neovascularization of adipose depots [88]. Moreover, miR-21 modulates components of the TGF-β signaling pathway, which are critically involved in adipose fibrosis and systemic insulin resistance [89]. Through these mechanisms, miR-21 integrates hypoxic stress with immunometabolic adaptation, reinforcing its relevance as a potential target in obesity-related inflammation and metabolic disease [90].

## The role of hypoxamiRs in cancer

Cancer cells frequently experience hypoxic stress due to rapid proliferation and insufficient vascularization. Rather than succumbing to oxygen deprivation, tumors reprogram hypoxia-responsive pathways, including hypoxamiRs (Table 2), to support angiogenesis, metabolic adaptation, immune evasion, and survival.

**Table 2:** Involvement of hypoxamiRs in cancer progression.

miRNA	Tissue/Context	Regulation under Hypoxia	Functions and Targets	Role in Cancer
**miR-210**	Breast, lung, colorectal, glioma	Upregulated	Promotes angiogenesis (via VEGF), regulates cell survival (via E2F3)	Supports tumor growth, angiogenesis, and resistance to apoptosis [91]
**miR-21**	Lung, breast, prostate, colon, pancreatic	Upregulated	Targets tumor suppressors (e.g., PTEN, PDCD4), pro-survival pathways (e.g., Bcl-2, IL-6)	Drives tumor cell survival, proliferation, and metastasis [128]
**miR-155**	Breast, lung, gastric, lymphoma	Upregulated	Regulates inflammation (via SOCS1, TNF receptor), immune responses	Contributes to tumor progression and immune evasion [129]
**miR-126**	Non-small cell lung cancer (NSCLC)	Upregulated	Targets negative regulators of angiogenesis (e.g., SPRED1, PIK3R2)	Enhances tumor vasculature, promotes cancer cell survival [130]
**miR-34a**	Colorectal, liver, breast, ovarian, lung	Downregulated	Targets p53 pathway, cell cycle regulators (e.g., CDK6)	Loss of function contributes to tumor progression and metastasis [131]
**miR-125b**	Breast, prostate, lung, ovarian	Upregulated	Inhibits apoptosis (via BCL2, caspase-3), promotes cell survival	Supports survival and proliferation of cancer cells [132]
**miR-143/145**	Colon, breast, prostate, lung	Downregulated	Inhibits cell proliferation (via K-Ras, ERK pathway)	Loss of miR-143/145 facilitates cancer progression and invasion [133]
**miR-96**	Ovarian, breast, gastric, colorectal	Upregulated	Targets tumor suppressors (e.g., PTEN), promotes cell cycle progression	Enhances proliferation and chemoresistance[134]
**miR-132**	Glioblastoma, lung, breast, neuroblastoma	Upregulated	Regulates cell growth and apoptosis (via p53, MAPK pathway)	Modulates tumorigenesis, survival, and migration[135]
**miR-424**	Glioblastoma, hepatocellular carcinoma	Upregulated	Enhances angiogenesis via CUL2 downregulation. stabilizes HIF-1α, promotes vascular remodeling	Correlates with therapy resistance and tumor vascularization in hypoxic tumors [104]
**miR-199a**	Liver, ovary, bladder	Downregulated	Targets HIF-1α and SIRT1, suppresses glycolysis and stress survival pathways	Enhances hypoxia-driven proliferation, metabolic reprogramming, and resistance to apoptosis [106]

These miRNAs contribute to essential tumorigenic processes, such as survival, angiogenesis, metastasis, and immune evasion, and they serve as potential therapeutic targets for treating hypoxia-related cancer progression.

### miR-210

miR-210 plays a multifaceted role in cancer by promoting angiogenesis [91], cellular survival, and metabolic adaptation [92], all of which enhance tumor growth and resistance to therapy in hypoxic environments [93]. In lung cancer, miR-210 supports cancer cell survival, promotes angiogenesis, and induces metabolic reprogramming, enabling the tumor to thrive under low oxygen conditions. miR-210 enhances tumor resilience by inhibiting apoptosis and stimulating glycolysis, making it a critical player in lung cancer progression. Moreover, its involvement in drug resistance further highlights its therapeutic potential, suggesting that targeting miR-210 could improve treatment outcomes [71].

In colon cancer, miR-210 stabilizes HIF-1 and promotes key processes such as angiogenesis, epithelial-to-mesenchymal transition (EMT), and metastasis. It is also implicated in chemotherapy resistance, emphasizing the need for novel therapeutic strategies. Under hypoxic conditions, colon cancer cells exhibit increased miR-210 and HIF-1α expression, promoting autophagy and reducing radiosensitivity through Bcl-2 downregulation [94]. Inhibiting miR-210 or HIF-1α reversed these effects, suggesting potential therapeutic targets. Additionally, PRP4 kinase, implicated in pre-mRNA splicing, promotes EMT and drug resistance by enhancing miR-210 transcription through HIF-1α-dependent mechanisms. Targeting PRP4 and the miR-210 axis could offer promising therapeutic strategies [95].

In triple-negative breast cancer (TNBC), miR-210 expression is upregulated under hypoxic conditions, contributing to cell proliferation, invasion, and metastasis. Elevated miR-210 levels in TNBC correlate with poor prognosis, positioning it as a potential prognostic marker and therapeutic target [96]. Its involvement in HIF-mediated signaling provides insight into potential treatment strategies, underscoring its significance in breast cancer progression. Pancreatic cancer, characterized by its aggressiveness and high mortality rate, also depends on hypoxia-induced miR-210 expression. MiR-210 promotes EMT, migration, and resistance to therapy by targeting genes such as EFNA3 and E2F3 [97]. Inhibiting miR-210 reduces hypoxia-driven malignancy, suggesting that targeting this miRNA could be an effective approach in treating pancreatic cancer. In hepatocellular carcinoma (HCC), miR-210 promotes metastasis and EMT, processes that contribute to the aggressive nature of HCC. Targeting miR-210 has been shown to enhance radiation sensitivity and apoptosis in HCC cells, presenting a promising therapeutic strategy, particularly in the context of hypoxia-induced resistance [98]. Neural brain tumors, including glioblastomas, often exist in hypoxic microenvironments, where miR-210 helps protect neural precursor cells from hypoxia-induced death by regulating BNIP3, a pro-apoptotic protein [99].

This dual role, promoting cell survival and resistance, illustrates the complex effects of miR-210 in brain tumor pathology. Inhibiting miR-210 may restore apoptosis and enhance therapeutic efficacy, offering a potential approach for improving treatment outcomes in hypoxic brain tumors [100].

In conclusion, miR-210 is a central mediator of hypoxia-induced signaling in cancer. Its role in tumor survival, metastasis, and therapy resistance positions it as both a biomarker and therapeutic target. As research continues, the therapeutic potential of targeting miR-210 across various cancer types may offer new strategies for improving patient outcomes and overcoming challenges associated with therapy resistance.

### miR-21

miR-21 is a well-characterized oncomiR whose expression is frequently upregulated under hypoxic conditions. It promotes cell survival by targeting pro-apoptotic genes such as programmed cell death 4 (PDCD4), phosphatase and tensin homolog (PTEN), and tropomyosin 1 (TPM1) [58]. In addition, miR-21 modulates immune checkpoint pathways and TGF-β signaling, facilitating immune evasion and tumor progression. Its expression is commonly elevated in breast, lung, colon, and pancreatic cancers, where it is associated with poor prognosis and therapy resistance [101].

### miR-155

miR-155 is another hypoxia-inducible miRNA that functions as an oncogenic regulator in various malignancies. It promotes inflammation, angiogenesis, and metastatic behavior, particularly in breast cancer, diffuse large B-cell lymphoma, and non-small cell lung cancer [102]. miR-155 targets multiple tumor suppressors including SOCS1 and SHIP1, thereby enhancing JAK/STAT signaling and sustaining an inflammatory tumor microenvironment. It is transcriptionally regulated by both HIF-1α and NF-κB and is considered a potential biomarker for cancer aggressiveness [103].

### miR-424

miR-424 is known to enhance angiogenesis in hypoxic tumors by targeting cullin 2 (CUL2), a component of the E3 ubiquitin ligase complex involved in HIF-1α degradation [104]. By downregulating CUL2, miR-424 contributes to HIF stabilization and promotes vascular remodeling. Elevated levels of miR-424 have been reported in gliomas and hepatocellular carcinoma, and correlate with resistance to anti-angiogenic therapies [104].

### miR-199

miR-199a, in contrast, is generally downregulated in hypoxia. It directly targets HIF-1α and SIRT1, both of which are crucial for the metabolic and survival adaptations of cancer cells in hypoxic niches [105]. Its silencing under hypoxic conditions leads to enhanced HIF signaling, metabolic reprogramming, and resistance to apoptosis. Loss of miR-199a has been observed in ovarian, liver, and bladder cancers, where it is associated with increased tumor aggressiveness [106].

## HypoxamiRs as diagnostic and prognostic biomarkers

HypoxamiRs are emerging as powerful non-invasive biomarkers, valued for their stability in body fluids, tissue-specific expression, and dynamic regulation under hypoxic stress [34]. For example, miR-210 is consistently elevated in breast and renal cancers, preeclampsia, and ischemic heart disease, with serum levels correlating with prognosis in clear cell renal carcinoma [107–109]. Similarly, miR-21 is upregulated in hypoxic tumor microenvironments and serves as a diagnostic marker in early-stage non-small cell lung cancer, as well as a tool for monitoring recurrence in colorectal cancer [110, 111].

Other notable candidates include miR-122, a liver-specific miRNA used to detect hypoxic injury and hepatotoxicity, and miR-126, a vascular marker implicated in diabetic complications and myocardial infarction [112]. Furthermore, miR-200c and miR-18a have shown promise as predictors of therapy response in breast and cervical cancers, respectively [113, 114]. Despite persistent technical challenges, including inter-platform variability, pre-analytical inconsistencies, and normalization difficulties, integrated hypoxamiR panels are gaining traction as tools for improved diagnosis, risk stratification, and treatment monitoring across hypoxia-related conditions.

## HypoxamiRs as therapeutic targets

Compared to conventional hypoxia-targeting strategies such as HIF inhibitors or anti-angiogenic agents, hypoxamiRs offer a more nuanced and physiologically attuned approach. As endogenous regulators finely tuned to oxygen levels and cellular context, they enable selective modulation of hypoxia-driven pathways without systemic suppression. Targeting hypoxamiRs allows modulation of coordinated gene networks involved in metabolism, angiogenesis, inflammation, and apoptosis, key processes in both adaptive and pathological responses to hypoxia. Moreover, their spatiotemporal expression patterns offer opportunities for precise, context-specific therapeutic interventions with reduced toxicity [26, 115, 116].

Preclinical studies have demonstrated the therapeutic utility of hypoxamiRs in both ischemic and oncologic models. miR-210, while not yet tested as a therapeutic in clinical trials, its biomarker role in ischemic stroke and bladder cancer is well-supported [107, 109, 117]. However, despite its rich mechanistic characterization, miR-210’s therapeutic development has lagged behind. Its pleiotropic and context-dependent effects pose significant challenges: while it promotes tissue repair in ischemia, its pro-survival and pro-angiogenic functions may exacerbate tumor progression. In addition, its broad target spectrum and ubiquitous expression complicate selective modulation, underscoring the need for delivery systems that ensure spatial and temporal precision. By contrast, oncogenic hypoxamiRs such as miR-21 and miR-155 have progressed further in translational pipelines. In preclinical cancer models, both have been successfully silenced using antagomiRs or LNA-modified oligonucleotides, resulting in tumor growth inhibition and enhanced treatment sensitivity [118]. Conversely, reintroduction of tumor-suppressive hypoxamiRs like miR-199a or miR-34a has been shown to downregulate HIF signaling and inhibit angiogenesis [119].

Effective delivery remains a major hurdle in miRNA therapeutics [120]. Hypoxia-sensitive nanoparticles, such as pH- or redox-responsive systems, have enabled tumor-specific delivery of miRNA modulators like miR-34a in preclinical lung cancer models [121]. Liposomal and aptamer-based carriers are also in development to enhance tissue specificity and minimize off-target effects.

Clinically, Cobomarsen (MRG-106), an inhibitor of miR-155, has completed phase I trials for cutaneous T-cell lymphoma and is being investigated in other hematologic malignancies [122]. Though MRX34, a miR-34a mimic, was discontinued due to immune-related toxicity, it marked the first systemic application of a miRNA therapeutic in oncology [121]. Future strategies are likely to involve combinations of hypoxamiR-targeted therapies with immunotherapies or anti-angiogenic agents to overcome treatment resistance and reshape hypoxic tumor responses.

Nonetheless, several translational challenges remain. These include the inherent instability of synthetic miRNAs, limited tissue specificity, immune activation, and inefficient intracellular delivery to hypoxic niches [123]. Additionally, the pleiotropic nature of miRNAs raises concerns about unintended off-target effects. Among all hypoxamiRs, miR-155 is currently the closest to clinical translation. Its targeted inhibition with Cobomarsen represents the most advanced therapeutic effort.

Together, these advances highlight both the potential and the complexity of leveraging hypoxamiRs for precision diagnostics and therapeutics in hypoxia-related diseases.

## Conclusion

Efforts to therapeutically target hypoxia, via HIF inhibitors, anti-angiogenic drugs, or metabolic modulators, have been constrained by systemic toxicity, incomplete specificity, and limited durability. HypoxamiRs represent a fundamentally different class of regulators: they are not just downstream markers of hypoxia but finely tuned sensors and effectors of oxygen-dependent gene regulation. Their context-specific expression, pleiotropic yet coordinated target networks, and inherent stability make them uniquely suited for both diagnostic and therapeutic applications.

This review argues that hypoxamiRs should be seen not simply as a catalog of hypoxia-regulated transcripts, but as a framework for understanding how cells interpret and respond to oxygen stress. Their dual capacity, as disease biomarkers and as levers of intervention, offers a route to precision medicine strategies that reflect the physiological complexity of hypoxic adaptation.

Nonetheless, key barriers remain. The challenges of miRNA delivery, tissue-specific targeting, immunogenicity, and off-target effects must be addressed through innovations in vector design, delivery platforms, and contextual functional analysis. As hypoxamiRs transition from molecular insight to clinical utility, their potential to shape future strategies for treating cancer, ischemia, fibrosis, and beyond becomes increasingly tangible.

If harnessed with precision, hypoxamiRs could shift the paradigm for managing hypoxia-related diseases, not by inhibiting the response, but by intelligently rewriting it.
